# Changes in the rhizosphere and root-associated bacteria community of white Guinea yam (*Dioscorea rotundata* Poir.) impacted by genotype and nitrogen fertilization

**DOI:** 10.1016/j.heliyon.2024.e33169

**Published:** 2024-06-15

**Authors:** Ayodeji Peter Idowu, Kosuke Yamamoto, Takahiko Koizumi, Minenosuke Matsutani, Kanako Takada, Yuh Shiwa, Asrat Asfaw, Ryo Matsumoto, Michel Ouyabe, Babil Pachakkil, Hidehiko Kikuno, Hironobu Shiwachi

**Affiliations:** a Department of International Agricultural Development, Faculty of International Agriculture and Food Studies, Tokyo University of Agriculture, Tokyo, Japan; b Department of Molecular Microbiology, Faculty of Life Sciences, Tokyo University of Agriculture, Tokyo, Japan; c NODAI Genome Research Center, Tokyo University of Agriculture, Tokyo, Japan; d International Institute of Tropical Agriculture (IITA), PMB 5320 Oyo Road Ibadan, Nigeria; e Miyako Subtropical Training and Research Farm, Tokyo University of Agriculture, Okinawa, Japan

**Keywords:** Bacterial community, 16S rRNA, Amplicon sequence variants, White Guinea yam, *Dioscorea rotundata* Poir, Symbiotic nitrogen-fixing bacteria

## Abstract

The bacterial diversity and composition of water yam (*Dioscorea alata* L. cv. A-19), which can grow without chemical fertilization, have recently been characterized with no significant differences compared with the use of chemical fertilization. However, the diversity and community structure of bacteria associated with the white Guinea yam *(Dioscorea rotundata*), the most cultivated and economically important yam in West Africa, have not yet been investigated. This study characterized the bacterial diversity and composition associated with bulk soil, rhizosphere, and plant roots in six white Guinea yam genotypes (S004, S020, S032, S042, S058, and S074) in field experiments in Ibadan, Nigeria under N-based chemical fertilizer application. The largest diversity of bacteria was found in the bulk soil, followed by the rhizosphere and roots. Based on the alpha diversity analysis, the bacterial diversity in both S020 and S042 increased with fertilizer application among the bulk soil samples. S058 grown under no-fertilizer conditions had the highest bacterial diversity among the rhizosphere samples. Beta diversity analysis highlighted the significant difference in the composition of bacteria associated with the genotypes and fertilizer treatments, and S032 had a unique bacterial composition compared to the other genotypes. The dominant phylum across all sample types was Proteobacteria. Actinobacteriota was the dominant phylum among bulk soil samples. At the genus level, *Bacillus* was the most abundant bacterial genus across both the control and treated samples. *Pseudomonas* was predominant across all rhizosphere samples. *Chryseobacterium*, *Sphingobium*, *Delftia* and *Klebsiella* associated with the rhizosphere were shown the altered relative abundance between the control and treated samples depending on genotypes. A genus related to symbiotic nitrogen-fixing bacteria, the *Allorhizobium-Neorhizobium-Pararhizobium-Rhizobium* clade, showed higher relative abundance among all root samples, indicating that it is a core bacterial genus. Furthermore, the field application of chemical fertilizer had a significant impact on the relative abundances of two genera related to symbiotic nitrogen-fixers, *Allorhizobium-Neorhizobium-Pararhizobium-Rhizobium* clade and *Bradyrhizobium* in the rhizosphere and root. These results suggest that N-based chemical fertilizers and plant genotypes would influence the compositional arrangement of associated bacterial communities, including symbiotic nitrogen-fixing bacteria.

## 1 Introduction

Yam (*Dioscorea* spp.), currently categorized as one of the top-ranked orphan crops [1], is a multi-species tuber crop belonging to the family *Dioscoreaceae* that is widely cultivated in Africa, Asia, parts of South America, the Caribbean, and the South Pacific Islands [2]. About 77 million metric tons of fresh yam tubers are produced annually worldwide, with Africa contributing approximately 98 % [3].

West Africa produces more than 90 % of the global yam and is generally regarded as the world's yam belt region [3]. In this region, white Guinea yam (*Dioscorea rotundata*), the predominant cultivar, plays a critical role in the human diet and sociocultural systems [2]. It is an essential ingredient in government policies on food security and poverty alleviation and serves as a source of foreign exchange and income generation for people in this region [4].

Interestingly, annual yam production over the last three decades surged at a growth rate of approximately 3.75 %. This growth is expected to increase over the next two decades by an additional 3 %, mainly from using landraces with fewer chemical inputs but expanded land cultivation. This practice, however, is no longer sustainable owing to global population increase and cattle ranching activities [4]. Meanwhile, the substantial surge in yam productivity when compared to other roots and tuber crops, particularly cassava and potatoes, over the years was only marginal [2,4]. Yam, by tradition, is a nutrient-loving plant generally referred to as “the first crop after bush fallow" due to its nutritional requirements [5]. However, yam yield and productivity in response to soil fertility vary from one genotype to another [6]. Furthermore, the available information on fertilizer use in yam cultivation is minimal and regionally based. Fertilizer recommendations for yam cultivation in Nigeria, however, vary among the regions and soil types [5,7,8]. These unresolved circumstances surrounding yam fertilization have spurred the global search for sustainable yam production [5]. Consequently, soil fertility and nutrient management globally is a major concern in modern agriculture, with nitrogen – an essential nutrient, being the most limiting factor [[9], [10], [11]] confronting the yield and productivity of many agricultural commodities, including yam [12].

Nigeria, the world's largest yam producer, relies heavily on fertilizers (64 %) for its yam production [5]. Contributing approximately 67 % of the total world yam production [3], yam fertilization in Nigeria varies based on land-use history and cultural practices, thus resulting in recommendations ranging from 200 kg/ha to 600 kg/ha across yam geographical zones [5]. Meanwhile, some yam varieties have previously been reported to thrive on low fertility, alkaline soil [[13], [14], [15], [16]]. These studies indicate a positive association between these varieties of yam and some suspected plant growth-promoting rhizobacteria (PGPR), which are known for their ability to perform nitrogen fixation, indole acetic acid (IAA) production, phosphate solubilization, and siderophores [[17], [18], [19]]. Similarly, many studies have shown that the application of N-based fertilizers increases yam productivity [20,21]. However, Kabeerathumma and Mohankumar [22] reported that the fertilizer response magnitude of yams varied with cultivar. In the same vein, George et al. [23] reported that nutrient uptake in yams depends on many factors, including plant root morphology, age, nutrient demand, supply, and utilization.

Blanket fertilizer recommendations, however, have led to increased production costs in some yam-producing countries, with apparent limitations on soil fertility [5,24]. Fertilizer recommendations in sustainable agriculture are therefore based on the soil nutrient status through soil testing and analysis, previous crop history, particularly nutrient needs, and estimated removal rates [5].

Findings from our previous studies highlighted the mechanisms of N absorption and yam responses to different fertilizer sources [17,18]. Rezaei et al. [14] reported that the dry weight of the aerial parts in treated plants was significantly higher compared to the control treatment, thus bolstering the claim of Suja [25], who reported higher aboveground growth and enhanced chlorophyll content in white Guinea yam under higher N application rates. Both authors in their report observed that the application of N-based fertilizer delayed tuber initiation and prolonged the yam-growing season. However, no significant difference was recorded in the growth parameters of water yam (*D. alata* L. cv. A-19) treated with or without N-based fertilizer, suggesting that the application of N-based fertilizer had no effect on the bacterial diversity and composition of water yam [26]. Meanwhile, some genera of symbiotic nitrogen-fixing bacteria (SNFB) within the clade of *Allorhizobium-Neorhizobium-Pararhizobium-Rhizobium* were found dominating the roots of water yam (*D. alata* L. cv. A-19). These findings imply that water yam can obtain nitrogen from the atmosphere through the activity of SNFB [15,26].

Despite being the most cultivated and economically important yam species in West Africa, there have been no studies on the bacterial diversity and community structure associated with the white Guinea yam (*D. rotundata*). Therefore, this study sought to investigate the effects of fertilizer application and genotype on the bacterial diversity and community structure associated with white Guinea yams using 16S rRNA metagenomic analysis.

This study is the first to report flora characterization of root- and rhizosphere-associated bacteria of white Guinea yam cultivated in the native field of the yam-producing country, Nigeria, comparing the community structure and diversity of the roots and rhizosphere-associated bacterial in six genotypes grown with or without NPK fertilizer.

## 2 Material and methods

### *2.1* *Plant materials and experimental desi*gn *for field experiments*

The six white Guinea yam genotypes (S004, S020, S032, S042, S058, and S074) were cultivated in the experimental field (N 7°30′, E 3°54′) of the International Institute of Tropical Agriculture (IITA, Ibadan), Nigeria, in 2018. These six genotypes are part of the mini-core collections of white Guinea yam and exhibit varying tuber yield and leaf density [6,27].

Matsumoto et al. [6] reported that these genotypes exhibited identical behavior in field-cultivated experiments and have been used to determine shoot and tuber biomass as well as nutrient use efficiency in white Guinea yam. Consequently, we also used these six genotypes in our study, and the experimental design is presented in Supplementary Table 1.

The field experiments followed the method described by Matsumoto et al. [6]. Fifty grams of freshly weighed yam seed, obtained from previously harvested yam tubers, was pre-sprouted in plastic pots filled with topsoil on the May 3, 2018. Upon sprouting, the yam seedlings were transferred to the IITA Ibadan research field (plot EE-24) on June 8, 2018.

Two treatments consisting of a control (no fertilizer) and different rates of NPK fertilizer (90 kg/ha N, 50 kg/ha P, and 75 kg/ha K), as recommended by Kayode [28] for yams planted on low-fertility soil, were used in this study. The physicochemical properties of the soil in this experimental field were previously described by Matsumoto et al. [6].

### 2.2 Sample collection and preparation

At 95–133 days after planting (DAP), below-ground (root and rhizosphere) samples were collected and cut into 4.5 cm long segments starting 1.0 cm below the root base. The bulk soil (BS) samples were collected from a depth of 1.0–5.5 cm from the soil surface, thus corresponding to a root length of 4.5 cm.

The root (R) samples were immersed in PBS-S for 20 min, shaken at 140 rpm on a rotary shaker to remove potential contaminants, and stored in a new sterile 50 ml falcon tube. The suspended solution was decanted to collect rhizosphere samples. Subsequently, the root samples were treated with 40 mL of 70 % ethanol for 2 min, followed by soaking in 40 ml of 1 % sodium hypochlorite for 5 min.

The root samples were rinsed five times with sterile water and gently dried with a paper towel. The suspended soil recovered from the root solution through centrifugation at 4000×*g* for 20 min at 25 °C was referred to as the rhizosphere (Rh) sample [29,30]. Subsequently, the BS, Rh and R samples were immersed in liquid nitrogen, transferred into a labeled airtight polythene bag, and stored at −80 °C until bacterial DNA extraction.

### 2.3 Extraction of bacterial DNA, PCR amplification, and Construction of 16S rRNA amplicon library

The root samples were ground to obtain a fine powder using a sterilized mortar and pestle and cooled using liquid nitrogen. For DNA extraction, 0.25 g of each of the rhizosphere, bulk soil, and powdered root samples was used.

Total DNA was extracted using the DNeasy PowerSoil Kit (QIAGEN) following the manufacturer's instructions. The accuracy of the extracted DNA concentration was verified using a Qubit dsDNA HS (High Sensitivity) assay kit (Thermo Fisher Scientific) according to the manufacturer's instructions.

To minimize the amplification of chloroplast and mitochondrial 16S rRNA gene during PCR [31], the V5–V7 region fragments were targeted and amplified using target-specific primer pairs of 799F, 5′-TCGTCGGCAGCGTCAGATGTGTATAAGAGACAGAACMGGATTAGATACCCKG-3′ and 1193R, 5′-GTCTCGTGGGCTCGGAGATGTGTATAAGAGACAGACGTCATCCCCACCTTCC-3′ with overhang adapters (shown as the underlined regions) of Illumina next-generation sequencing for the amplification of the V5–V7 region fragments of the 16S rRNA prokaryotic gene [32,33].

The generated PCR amplicon library was prepared according to the guidelines of the Illumina 16S Sample Preparation Guide (15044223-b; Illumina, Inc.). We assessed the quality of the library using a D1000 Screen Tape on the Agilent 4200 TapeStation (Agilent Technologies, Inc.) and sequenced it as a paired-end sequence of 300 bp using an Illumina MiSeq sequencing platform.

### 2.4 Processing and analysis of the obtained sequence

The obtained sequences were analyzed using the protocol described by Zabat et al. [34]. Raw paired-end FASTQ files were filtered, trimmed, denoised, and merged using the QIIME 2 Divisive Amplicon Denoising Algorithm 2 (DADA2) plug-in. We generate clean sequences using the 2018 version 11 of the Quantitative Insights Into Microbial Ecology 2 (QIIME2) program [35] following the method described by Callahan et al. [36].

The amplicon sequence variants (ASV) were clustered at 100 % identity. The removal of the identified chimeric sequences was done using the consensus method in the DADA2 program. The QIIME 2 feature-classifier plugin utilized the pre-trained Naïve Bayes classifier on SILVA (ver. 138) to trim the ASV database to the V5–V7 region.

The obtained 16S rRNA gene sequences were taxonomically classified using the method described by DeSantis et al. [37]. Multiple sequence alignment, diversity metric calculations, and phylogenetic reconstruction were performed using MAFFT, QIIME2 diversity plug-in and FastTree, respectively [38,39].

Three samples, S004-BS-C-2, S074–Rh–C-1, and S074–Rh–C-2 were excluded from our statistical analyses due to insufficient reads (Supplementary Table 2).

### 2.5 Statistical data analysis

Using version 3.4.3 of R, we subsequently analyzed the phylogenetic community structures using the packages phyloseq [40] and pheatmap [41], followed by normalization after filtration of the classified chloroplast and mitochondrial ASVs. Diversity index differences, including alpha diversities (Richness, Shannon, and Simpson indices), were checked using a one-way analysis of variance (ANOVA).

Based on the estimated total abundance matrix, the interactions within and between the bacterial community structures were determined according to non-metric multidimensional scaling (NMDS) of Bray–Curtis dissimilarity and phylogenetic β-diversity metrics (abundance-weighted UniFrac distance).

The FDR-adjusted p-value was by default, set to a cut-off of 0.1 and the log LDA to 2.0, following the method by Dhariwal et al. [42]. The LDA Effect Size (LEfSe) submodule within Microbiome Analyst (https://www.microbiomeanalyst.ca/) was then calculated.

## 3 Results

### 3.1 General analyses of the sequenced data

A total of 3,111,128 high-quality non-chimeric sequence reads were obtained after the filtration and normalization of 8,221,601 raw reads from the MiSeq sequencing analysis of 108 samples (Supplementary Table 2).

We obtained a total of 46,616 ASVs ranging between 4364 and 202 ASVs per sample except for S004-BS-C-2, S074–Rh–C-1, and S074–Rh–C-2 (Supplementary Table 2). As shown in Supplementary Fig. 1, the alpha rarefaction curve of the ASVs from each sample was almost saturated; therefore, most of the bacterial groups were detected.

### 3.2 Diversity of bacteria associated with genotypes and nitrogen fertilization

**Fig. 1:**
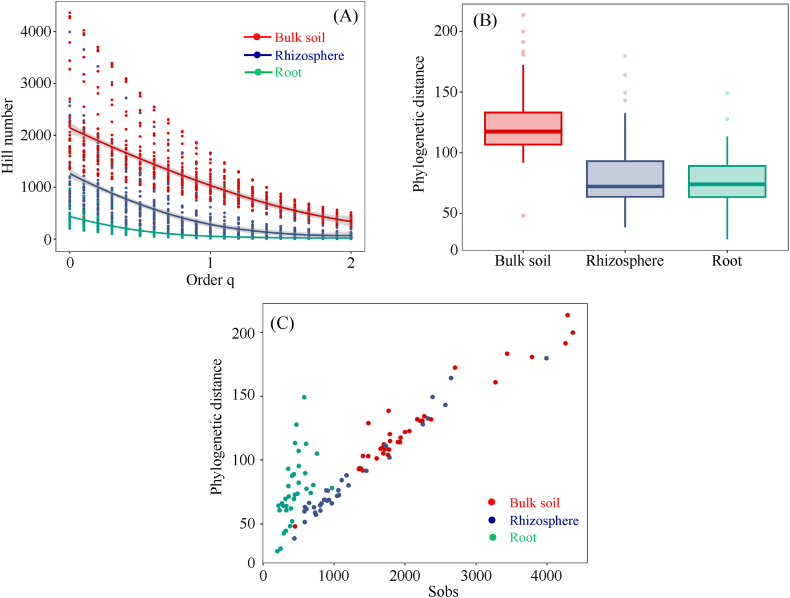
Comparison of bacterial diversity among bulk soil, rhizosphere, and root samples from six genotypes of white Guinea yam cultivated in an agricultural field in Nigeria. Bulk soil, rhizosphere, and root samples are shown in red-, blue-, and green-colored dots, respectively. (A) A diversity profile curve plotting Hill numbers as a function of order q (q = 0, species richness; q = 1, Shannon diversity; q = 2, Simpson diversity). Data are shown in Supplementary Table 3. The 95 % confidence intervals (shaded areas) were obtained by a bootstrap method based on 200 replications. (B) Comparison of phylogenetic distances among bulk soil, rhizosphere, and root samples. (C) Correlation analysis between phylogenetic distance and species richness.

A comparison of the Hill number profiles and phylogenetic distances among the three sample types showed that the bulk soil had the highest bacterial diversity, followed by the rhizosphere and roots (Fig. 1A and B).

Correlation analysis between the phylogenetic distance and the observed ASVs exhibited a bimodal correlation, where the root samples had a greater phylogenetic distance despite having fewer observed ASVs than the rhizosphere and bulk soil samples. This result indicates that root bacterial diversity in white Guinea yam could be maintained by selectively permitting root inhabitation of limited bacterial species with various phylogenetic distances (Fig. 1C).

**Fig. 2:**
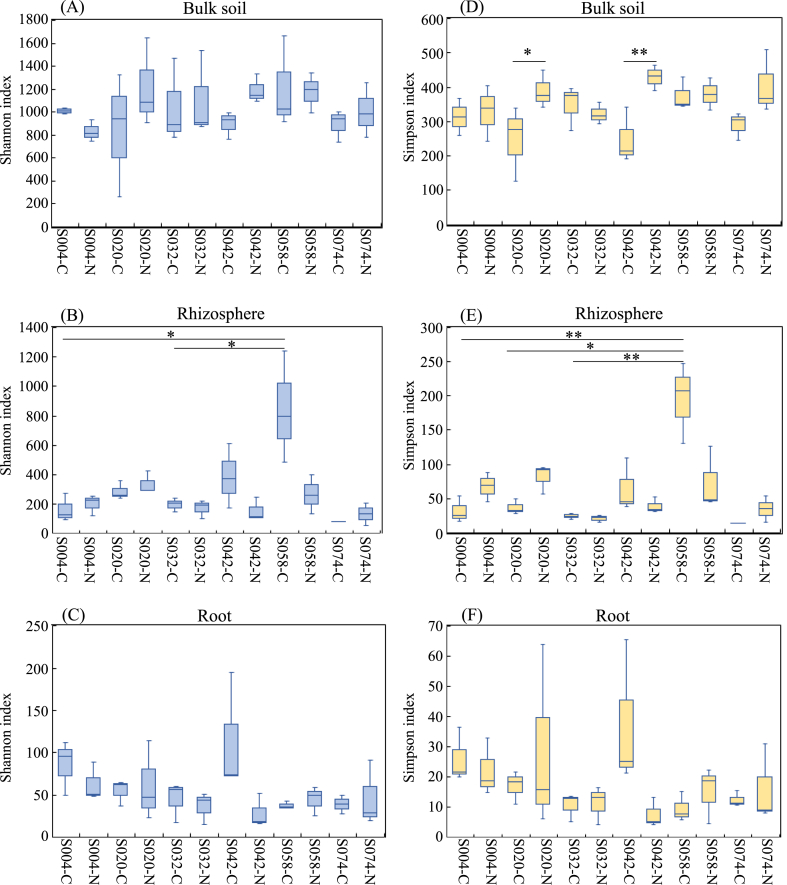
Alpha diversity indices for the 16S rRNA gene sequences. Box plots of the observed Shannon (A–C) and Simpson (D–F) indices in bulk soil, rhizosphere, and root samples from six genotypes of white Guinea yam cultivated with or without chemical fertilizer input. Whiskers represent the minimum and maximum values. All other points are contained within the box, and the bar represents the median. Statistical analysis of alpha diversity data was performed using ANOVA and the Tukey-Kramer test. Asterisks indicate statistically significant differences between pairs of values (*P < 0.05, **P < 0.01). C, non-fertilizing control; N, chemical fertilizer treatment.

The alpha diversity metrics showed a significant difference (P < 0.05) in the Simpson indices among bulk soil samples for S020 (df = 5, P = 0.0473) and S042 (df = 5, P = 0.0012) with and without fertilizer, respectively (Fig. 2D), although there was no significant difference in the Shannon indices in bulk soil samples (Fig. 2A).

Among the rhizosphere samples, the highest bacterial diversity was obtained in the control sample of S058, thus exhibiting a significantly higher Shannon index with a p-value of 0.0215 (df = 5), compared with the control samples of S004 and S032 with a p-value of 0.0387 (df = 5) (Fig. 2B). The same applied to the Simpson index (df = 5 each, P = 0.0066 against S004–C, P = 0.0101 against S020–C, and P = 0.0030 against S032–C) (Fig. 2E).

However, there was no significant difference in the bacterial diversity of the root samples among the genotypes and fertilizer treatments for either index (Fig. 2C–F). Furthermore, the coverage index of each sample group was over 99 %, suggesting that the data accurately reflected bacterial diversity (Supplementary Table 3). These findings are consistent with the alpha-rarefaction curve results, as shown in Supplementary Fig. 1.

### 3.3 Comparative analysis of bacterial communities associated with genotypes fertilizer treatments

**Fig. 3:**
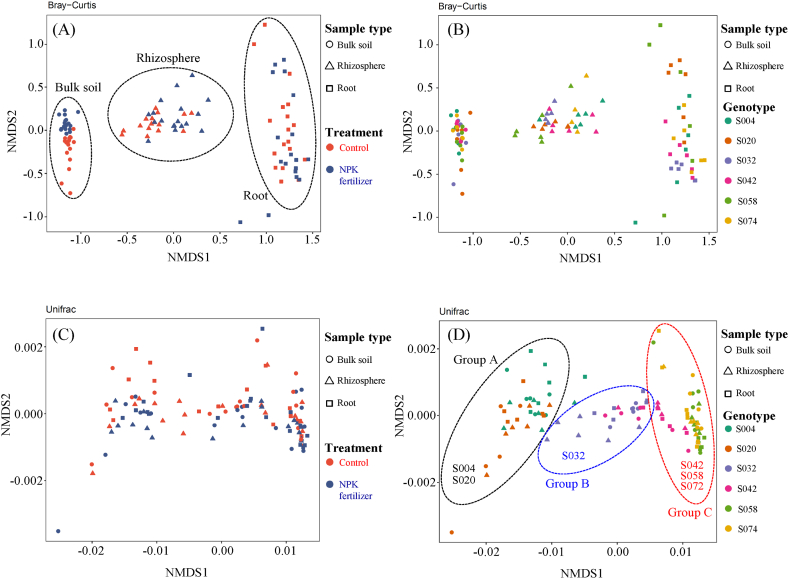
Non-metric multidimensional scaling (NMDS) analysis of the bacterial communities in different samples based on Bray-Curtis distances (A, B) and weighted UniFrac distances (C, D). Sample types are classified based on different shapes; circle, triangle, and square represent bulk soil, rhizosphere, and root, respectively. The differences between treatment with or without NPK fertilizer (A and C) and genotypes (B and D) are divided by color codes, as described in the explanatory notes within each panel.

NMDS and PERMANOVA calculated by Bray-Curtis distance indicated a clear separation between the bacterial communities among the sample types (PERMANOVA, R2 = 0.383, Pr (>F) = 0.0001), genotype (PERMANOVA, R2 = 0.055, Pr (>F) = 0.0009) and fertilizer treatment (PERMANOVA, R2 = 0.021, Pr (>F) = 0.0009), but not by sampling day (PERMANOVA, R2 = 0.005, Pr (>F) = 0.5309) (Fig. 3A and B and Supplementary Table 4).

PERMANOVA analysis revealed that sample type was the most important factor in separating bacterial communities. In bulk soil samples, fertilizer application significantly influenced the composition of the bacterial communities (PERMANOVA, R2 = 0.115, Pr (>F) = 0.0001).

This result indicates that the communities of bacterial associated with bulk soil were unaffected by differences in genotypes, thus confirming accurate sampling from each field. PERMANOVA testing indicated that plant genotype appeared to be the most important factor in differentiating bacterial communities in both root and rhizosphere samples, followed by fertilizer treatment (Supplementary Table 4).

The NMDS based on the weighted UniFrac distance seemed to create three clusters according to the genotypes described in Fig. 3D as groups A-C (Fig. 3C and D). The calculated weighted UniFrac distance where the most contributing factor in separating the bacterial communities was the plant genotypes (PERMANOVA, R2 = 0.88741, Pr (>F) = 0.0001), followed by fertilizer treatment (Supplementary Table 5).

The observed clustering pattern was confirmed by PERMANOVA testing, demonstrating that the bacterial community structure in the six white Guinea yam genotypes clustered according to genotype.

### 3.4 Microbial Taxonomic analysis at the phylum level

**Fig. 4:**
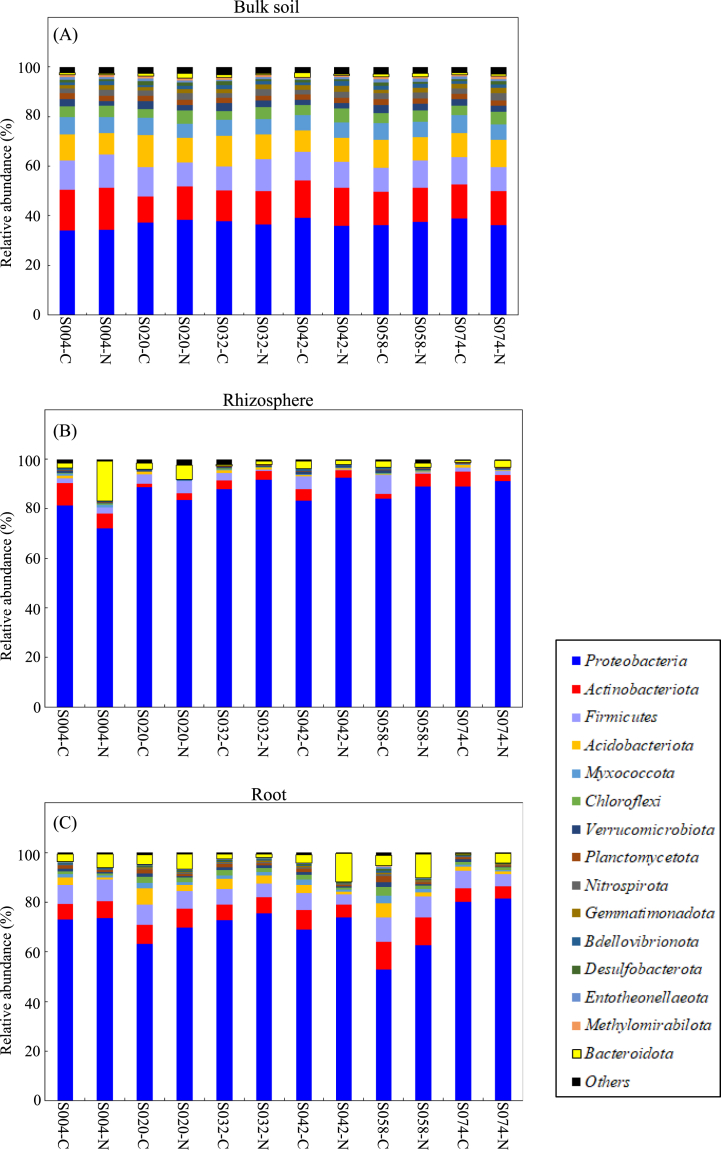
Average relative abundances of bacteria at the phylum level in bulk soil (A), rhizosphere (B), and root (C) samples. C, non-fertilizing control; N, chemical fertilizer treatment.

High-quality sequence classification at the phylum level elucidated bacterial community differences based on plant genotypes and fertilizer treatments. Forty-four phyla, including one yet-to-be-identified phylum, were found in all the samples. The relative abundances of the top 15 phyla are shown in Fig. 4 and Supplementary Table 6.

Proteobacteria was the most dominant phylum across all samples used in this study, accounting for approximately 34–92 %. The dominant phylum in the bulk soil samples was Actinobacteriota, constituting over 10 % of the relative abundance, whereas Firmicutes, Acidobacteriota, and Myxococcota were the sub-dominant phyla, accounting for more than 5 %.

Bulk soil samples of S020, S042, and S074 collected from fields treated with N-based fertilizer contained Chloroflexi as their sub-dominant phyla. Meanwhile, the rhizosphere samples of the treated field, excluding S042 and S074, contained Actinobacteriota and Firmicutes as the sub-dominant phyla.

S058 had Actinobacteriota as the dominant phylum, constituting approximately 11 % of the total. Acidobacteriota in S020–Rh and S058–Rh decreased in relative abundance from 6 % to 2 % in fields treated with chemical fertilizer. In contrast, Bacteroidota in the rhizosphere samples of S004, S020, S042, and S058 increased to the level of the dominant phyla under the chemical fertilizer treatment.

The same was true for the root samples of S004 and S020. Meanwhile, in the root samples of S042 and S058, the relative abundance of the associated Firmicutes diminished to less than the sub-dominant phylum under the chemical fertilizer treatment.

### 3.5 Structural classification of the community of bacteria genus

**Fig. 5:**
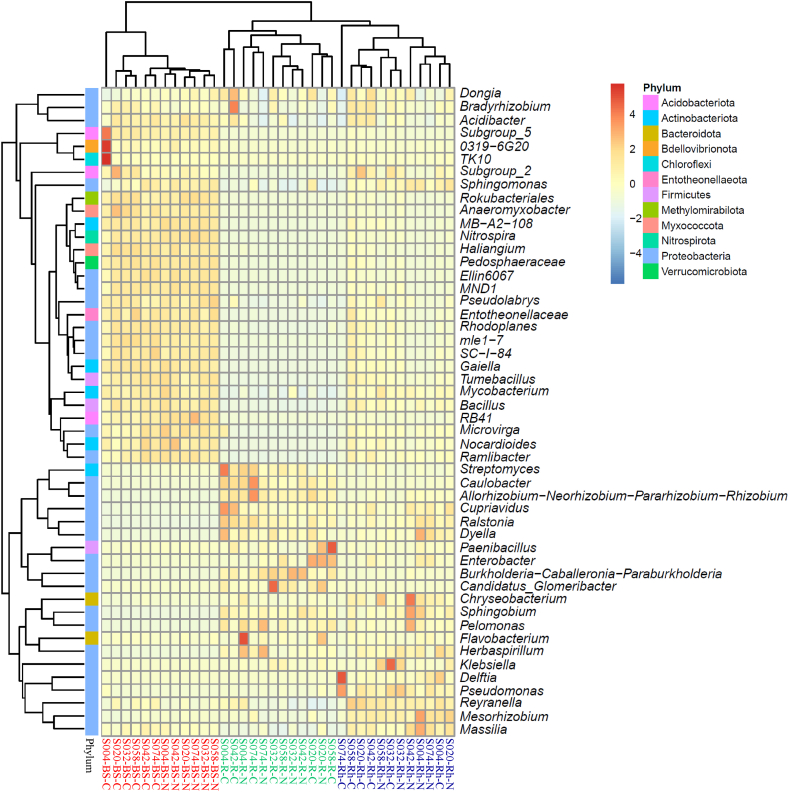
Heatmap of the bacterial distribution of the top 50 abundant genera in all sample types. The dendrogram shows complete-linkage agglomerative clustering based on Euclidean distance. The heatmap color (blue to red) represents the row *z*-score of the mean relative abundance from low to high. BS, bulk soil; R, root; Rh, rhizosphere; C, non-fertilizing control; N, chemical fertilizer treatment.

Observational analysis of the top 50 identified genera revealed that the bacterial genera were classified and clustered under 12 bacterial phyla based on a heatmap analysis (Fig. 5 and Supplementary Table 7). Among the observed bacterial genera in bulk soil samples, *Bacillus* was the most abundant bacterial genus, dominantly constituting approximately 5.8 %–9.7 % of both the control and treated samples.

Four genera (*Subgroup 5*, *0319-6G20*, *TK10*, and *Pseudomonas*) were highly abundant in the control sample of S004. However, in the rhizosphere samples, *Pseudomonas* was predominant, spreading from approximately 4.9 %–43.5 % across both treatments (treated and untreated).

*Chryseobacterium* associated with the rhizosphere of S004, S042, and S058 increased in relative abundance with fertilizer application, similar to *Sphingobium* in the rhizosphere of S042. Chemical fertilizers negatively affected the relative abundance of *Delftia* in the rhizosphere of S004 and S074. Similar results were obtained in the rhizosphere of S032 for *Klebsiella* under the same conditions.

A genus related to the symbiotic nitrogen-fixing bacteria (SNFB), *Allorhizobium-Neorhizobium-Pararhizobium-Rhizobium* clade, showed a higher relative abundance (>10 % of the total root sample relative abundance). In addition, two genera related to SNFB, *Bradyrhizobium* and *Mesorhizobium*, were found across all root samples, ranging from 6.1 % to 0.6 % and 0.5 %–0.1 %, respectively.

The relative abundance of *Streptomyces* inhabiting the roots of S004, S042, and S074 decreased under the chemical fertilizer treatment. The same applied to *Culobacter* in S074 and *Candidatus_Glomeribacter* in S032 under the same conditions. In contrast, the application of chemical fertilizer in the fields positively influenced the relative abundance of *Flavobacterium* associated with the roots of S004 and S020.

These results suggest that both N-based fertilizer application and plant genotype influenced the distribution and relative abundance of bacterial genera associated with white Guinea yam plants.

### 3.6 Statistically significant microbial communities at genus level between treated and untreated with chemical fertilizer within a sample type

**Fig. 6:**
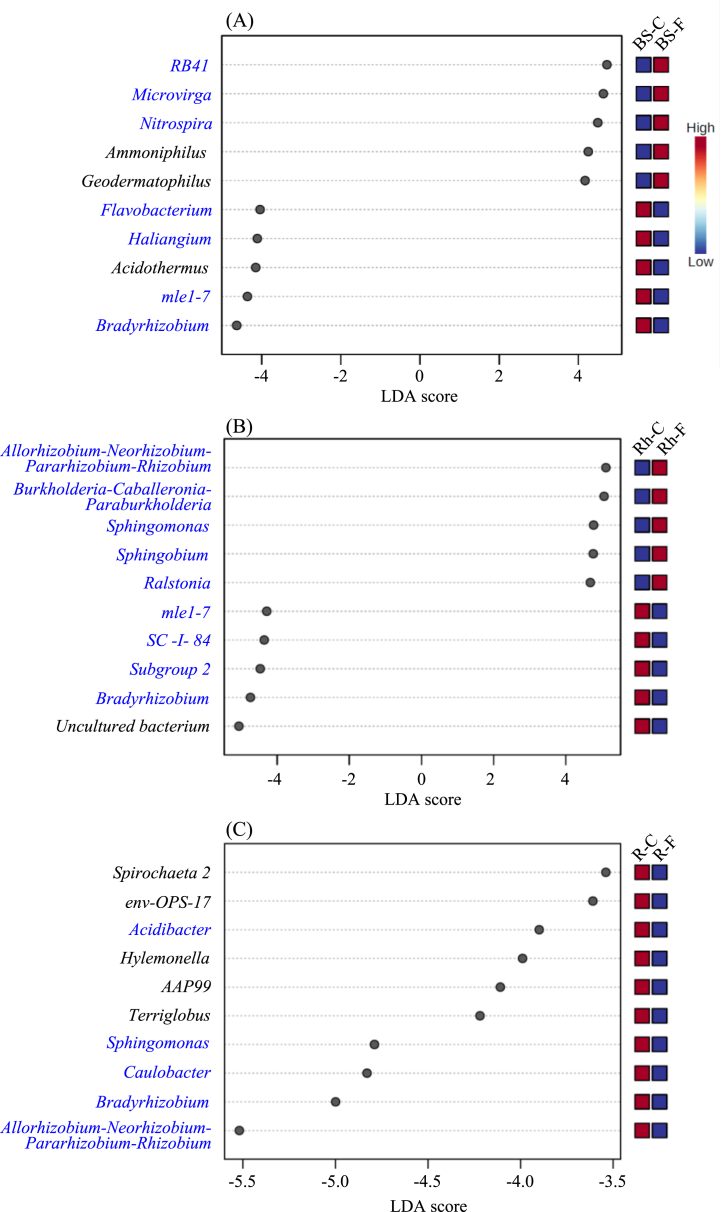
LEfSe analysis of bacterial genera (top 10) in bulk soil (A), rhizosphere (B), and root (C) of white Guinea yam between treatment with and without chemical fertilizer inputs. LDA scores of biomarker bacteria. LDA scores are horizontal bars for the biomarker bacteria with an LDA score >2.0. The positive LDA scores indicate the enriched genera in each sample type by treatment. In contrast, significantly diminished genera are represented on the negative LDA scores in each sample type. The genera colored in blue are also shown in heatmap data as the top 50 abundant genera in all sample types (Fig. 5). BS, bulk soil; R, root; Rh, rhizosphere; C, non-fertilizing control; N, chemical fertilizer treatment.

Based on LEfSe analysis, the top 10 unique (LDA score >2.0 and FDR-adjusted P-value <0.1) genera identified specialized as communities between the treated and untreated samples in this study (Fig. 6).

*RB41*, *Microvirga*, *Nitrospira*, *Ammoniphilus*, and *Geodermatophilus* were significantly enriched, whereas *Flavobacterium*, *Haliangium*, *Acidothermus*, *mle1-7*, and *Bradyrhizobium* were remarkably reduced in bulk soil samples treated with chemical fertilizer (Fig. 6A). In addition, chemical fertilization significantly altered the structure of the bacterial community in the rhizosphere and roots at the genus level (Fig. 6B and C).

**Fig. 7:**
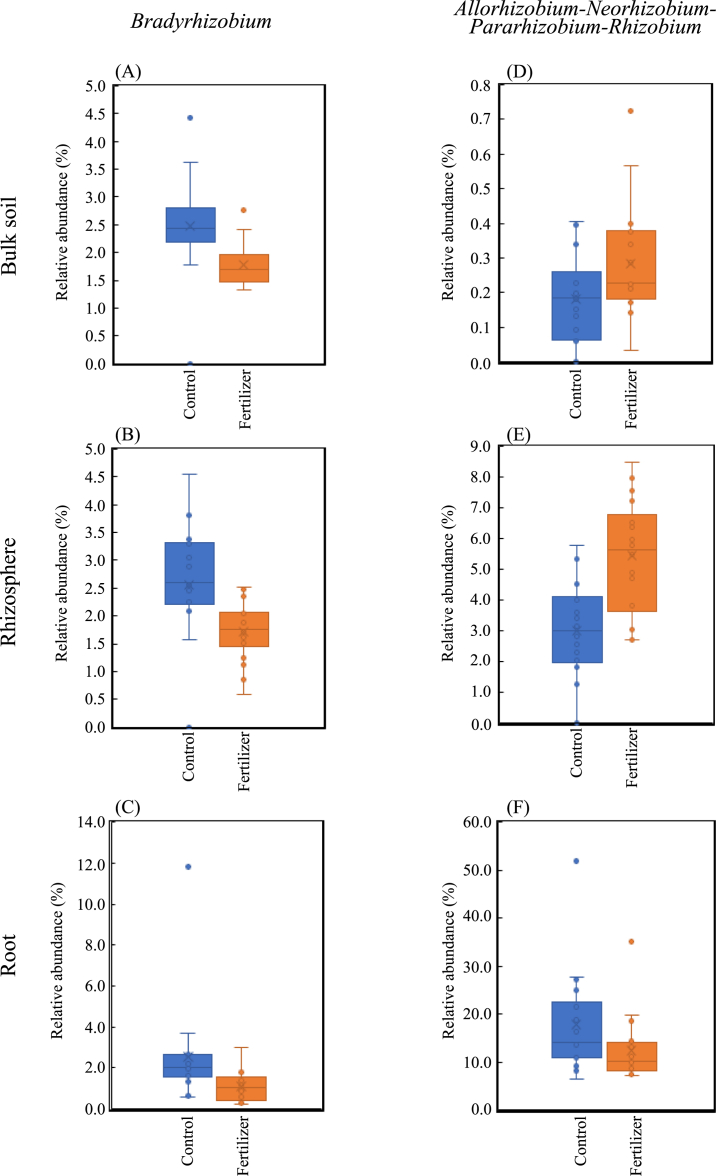
Changes in the relative abundance of two genera related to symbiotic nitrogen-fixing bacteria, *Bradyrhizobium* and *Allorhizobium-Neorhizobium-Pararhizobium-Rhizobium*, in white Guinea yam treated with chemical fertilizer. (A–C) Relative abundance of *Bradyrhizobium* in bulk soil, rhizosphere, and root samples. (D–F) Relative abundance of *Allorhizobium-Neorhizobium-Pararhizobium-Rhizobium* in bulk soil, rhizosphere, and root samples.

With a specific emphasis on the SNFB, the *Allorhizobium-Neorhizobium-Pararhizobium-Rhizobium* clade significantly increased, whereas *Bradyrhizobium* remarkably decreased in the rhizosphere under chemical fertilizers (Fig. 6B). Similar results were obtained for the relative abundance of both the bulk soil and rhizosphere-associated *Allorhizobium-Neorhizobium-Pararhizobium-Rhizobium* clade, and *Bradyrhizobium* (Fig. 7A, B, D, and E).

Meanwhile, all ten bacteria genera were significantly reduced in root samples treated with chemical fertilizer. Interestingly, among the bacteria genera negatively impacted by chemical fertilization in roots samples were two nitrogen-fixing bacteria-related genera *Allorhizobium-Neorhizobium-Pararhizobium-Rhizobium* clade and *Bradyrhizobium* (Fig. 6, Fig. 7F).

These results suggest that these bacterial genera exhibit different behaviors under different treatments (with or without chemical fertilizer). It is worth noting that the *Allorhizobium-Neorhizobium-Pararhizobium-Rhizobium* clade was released from the roots and attracted to the rhizosphere.

The proliferation ability of *Bradyrhizobium* was significantly lower in bulk soil under chemical fertilizer treatment, thus explaining its decreased abundance in both the rhizosphere and root samples.

## 4 Discussion

Our study investigated the effects of N fertilizer application on the composition and diversity of the bacterial community associated with white Guinea yam (*D. rotundata*) using 16S rRNA gene amplicon-based metagenomics, a culture-independent approach. The diversity of the bacterial community among the selected sample types was in the descending order of bulk soil > rhizosphere > root based on Hill number profiles and phylogenetic distance. The obtained result was similar to those of previous reports, where the reported bacterial diversity gradually decreased in pecking order from the soil to the root endosphere, and the rhizosphere had higher diversity than the root samples [[43], [44], [45], [46], [47]]. Although the lowest phylogenetic distances were recorded in the root samples of the plant genotypes used in the present study. The increasing rate of phylogenetic distance corresponding to the number of observed ASVs was more significant than that in the bulk soil and rhizosphere samples. This is because bacteria inhabiting the rhizosphere and root endosphere are directly affected by the host genotype and immune system, thereby shifting the bacterial composition from an enriched soil-derived community to a reduced host-adapted bacterial community [47]. A balance in the diversity of soil microbes is a critical factor in determining plant health [48], and diversity depends on the resistance of the microbial community to pathogen invasion [49]. Therefore, the limited bacterial species richness in the root, which bears a rich phylogenetic distance, contributes to the maintenance of bacterial diversity and supports the root health of white Guinea yam.

Alpha diversity analysis showed no significant differences in the diversity indices of the bulk soil samples among the plant genotypes, indicating that the field experiment was accurately performed, thereby maintaining the bacterial community variation to a minimum fraction among the testing sites. This result supports the beta diversity data calculated by the Bray-Curtis distance, where plant genotypes had no significant influence on the structure of the bacterial community in bulk soil samples. There were substantial differences in the diversity indices of the rhizosphere samples among genotypes. Similarly, there were no differences among the root sample genotypes or between the control and treated samples of one genotype. Yuying et al. [50] reported similar results, where applying a chemical fertilizer (NPK) did not significantly affect the alpha diversity (species richness and phylogenetic diversity) of endophytic bacteria on the roots of a wheat crop. Furthermore, the effect of plant genotype on root alpha diversity associated with *Boechera stricta* compared to leaf samples [51] was weak. These results indicate that the diversity of root-associated bacteria in white Guinea yam was robust across both genotypes and fertilizer treatments. Conversely, the analysis of beta diversity using both Bray-Curtis and weighted UniFrac distances revealed that the two factors (plant genotype and fertilizer treatment) significantly influenced the community structure of bacteria associated with bulk soil, rhizosphere, and roots, which is in line with the data from Fig. 4, Fig. 5; that is, the relative abundance of phyla and genera was affected by both fertilizer treatment and genotypes.

Any changes in the soil microbiome caused by fertilizer application can lead to changes in the bacterial community structure of the root endosphere [52]. Many studies have also demonstrated the impact of different fertilizer treatments on the community composition and structural arrangement of bacteria in the sugarcane rhizosphere [53] and wheat roots [50]. In contrast, Kihara et al. [26] reported that the application of chemical N fertilizer had no significant influence on the bacterial community associated with water yam cv. A-19, suggesting that the susceptibility of the bacterial community to soil NPK differs and reflects the species differences between water yam (*D. alata*) and white Guinea yam (*D. rotundata).* The distribution patterns of the NMDS plots calculated with weighted UniFrac distances seemed to separate the three groups, A, B, and C (Fig. 3), depending on the genotype, thus indicating that host genotypes would affect the structures of the bacterial community associated with white Guinea yam.

Aira et al. [54] reported similar results in which two genotypes (*su1* and *sh2*) of maize plants determined the different microbial communities in their rhizospheres. Previous studies on the two yam genotypes, S004 and S020 (group A in Fig. 3), demonstrated their susceptibility to soil NPK conditions, leading to increased shoot and tuber biomass under N-based fertilization [6]. In contrast, the yam genotype S032 (belonging to group B in Fig. 3) showed less fluctuation in aboveground (shoot) and belowground (tuber) production between the control and fertilizer treatments, indicating that S032 is a less susceptible genotype and could serve as potential parent stock for the generation of a varietal candidate for immediate release owing to its stable yield, less sensitivity to soil fertility conditions, or a new variety for low soil fertility in West Africa [6]. Consequently, variations in the bacterial community structure depending on the host genotype could be related to genotypic variation in the use and uptake efficiency of soil-available nutrients. Many studies have reported the isolation, identification, and estimation of nitrogen-fixing bacteria in non-leguminous crops, particularly in crops like sweet potatoes [55], sugarcane [56] and rice [57].

We reported that SNFB associated with the yam plants *D. esculenta* and *D. alata* play an important role in growth on non-fertile soils by fixing atmospheric nitrogen [13,14,[16], [17], [18], [19]]. In this study, we observed an extremely high (>10 %) relative abundance of *Allorhizobium-Neorhizobium-Pararhizobium-Rhizobium* clade across root samples in all genotypes.

*Bradyrhizobium* and *Mesorhizobium* were also found in the roots, although the relative abundances of both genera were lower than those of *Allorhizobium-Neorhizobium-Pararhizobium-Rhizobium*. The genera *Allorhizobium-Neorhizobium-Pararhizobium-Rhizobium* and *Mesorhizobium* were typically referred to as “fast-growing rhizobia," while *Bradyrhizobium* is known as “slow-growing rhizobia" [58,59].

Groundnut (*Arachis hypogea* L.), a leguminous plant, is strongly associated with either fast- or slow-growing rhizobia of the genera *Rhizobium* and *Bradyrhizobium* respectively [60,61]. At least three genera related to SNFB found inhabiting the root hairs of white Guinea yam could serve as potential ingredients for developing biological nitrogen fixation in their contributing order: *Allorhizobium-Neorhizobium-Pararhizobium-Rhizobium* clade > *Bradyrhizobium* > *Mesorhizobium*, following the results of their relative abundances.

Interestingly, two SNFB-related genera, the *Allorhizobium-Neorhizobium-Pararhizobium-Rhizobium* clade and *Bradyrhizobium*, were significantly lower in the root samples from fields with chemical fertilizer treatment.

The genus *Allorhizobium-Neorhizobium-Pararhizobium-Rhizobium* was attracted, and *Bradyrhizobium* was reduced in the rhizosphere area, suggesting that white Guinea yam may not necessarily establish a symbiotic relationship with SNFB because they can take up N derived from chemical fertilizers. Reid et al. [62] found that the population of rhizobacteria that could benefit plants through the mobilization of insoluble nutrients in the soil was lower under chemical fertilization.

**Fig. 8:**
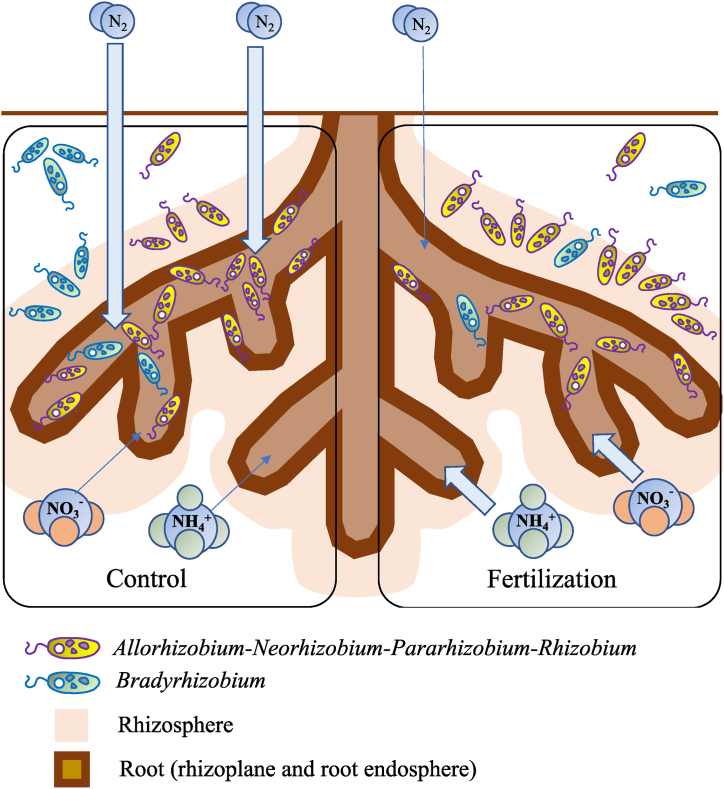
Schematic of our hypothesis for our future study; alteration of nutrient uptake and habitat for *Bradyrhizobium* and *Allorhizobium-Neorhizobium-Pararhizobium-Rhizobium* in the fields untreated and treated with chemical fertilizer.

These results enable us to construct our future hypothesis that white Guinea yam-associated SNFB in nutrient-deficient soil is responsible for the stable growth of both belowground and aboveground parts, as shown in Fig. 8 because white Guinea yam could take up N directly as NH_4_^+^ is converted from N_2_ in the atmosphere. In other words, the growth of white Guinea yam in fields with sufficient NPK input could enhance the uptake of N derived from chemical fertilizers rather than biologically fixed N.

## 5 Conclusions

This is the first report to characterize the bacterial community of white Guinea yam cultivated with and without N-based chemical fertilizer. Our study suggests that (i) the bacterial community structure associated with white Guinea yam was significantly affected by plant genotypes and fertilizer application, and (ii) the relative abundances of two SNFB-related genera, the *Allorhizobium-Neorhizobium-Pararhizobium-Rhizobium* clade and *Bradyrhizobium* were significantly decreased in the root samples under mineral fertilization.

This study provides new insights into breeding programs aimed at improving white Guinea yam varieties. In a subsequent study, to address our hypothesis (Fig. 8), we will characterize the N isotope (δ15 N) absorption ratio and bacterial communities associated with several varieties with stable productivity under low soil fertility conditions to clarify whether there is a relationship between bacterial communities, including SNFB, and stable productivity under various exogenous soil environments.

## Data availability statement

We have deposited all the obtained sequence data in this study in the DDBJ Sequence Read Archive (DRA) database as follows: (1) accession numbers, DRA016644; BioProject, PRJDB16178; BioSample, SAMD00629971-SAMD00630078; (2) accession numbers, DRA016645; BioProject, PRJDB16178; BioSample, SAMD00630079-SAMD00630136.

## CRediT authorship contribution statement

**Ayodeji Peter Idowu:** Writing – review & editing, Writing – original draft, Investigation, Funding acquisition, Formal analysis, Data curation. **Kosuke Yamamoto:** Writing – review & editing, Writing – original draft, Validation, Supervision, Methodology, Formal analysis, Conceptualization. **Takahiko Koizumi:** Visualization, Formal analysis. **Minenosuke Matsutani:** Formal analysis. **Kanako Takada:** Data curation. **Yuh Shiwa:** Formal analysis. **Asrat Asfaw:** Resources, Formal analysis. **Ryo Matsumoto:** Resources, Formal analysis. **Michel Ouyabe:** Formal analysis. **Babil Pachakkil:** Formal analysis. **Hidehiko Kikuno:** Formal analysis. **Hironobu Shiwachi:** Supervision, Project administration, Funding acquisition, Conceptualization.

## Declaration of competing interest

The authors declare that they have no known competing financial interests or personal relationships that could have appeared to influence the work reported in this paper.
