# Supplementary material for: Changes in the rhizosphere and root-associated bacteria community of white Guinea yam (Dioscorea rotundata Poir.) impacted by genotype and nitrogen fertilization
Source: Heliyon. 2024 Jun 15;10(12):e33169. doi: 10.1016/j.heliyon.2024.e33169 (PMC11252748; doi:10.1016/j.heliyon.2024.e33169)
Supplement: Multimedia component 1 [file mmc1.pptx]

## Slide 1
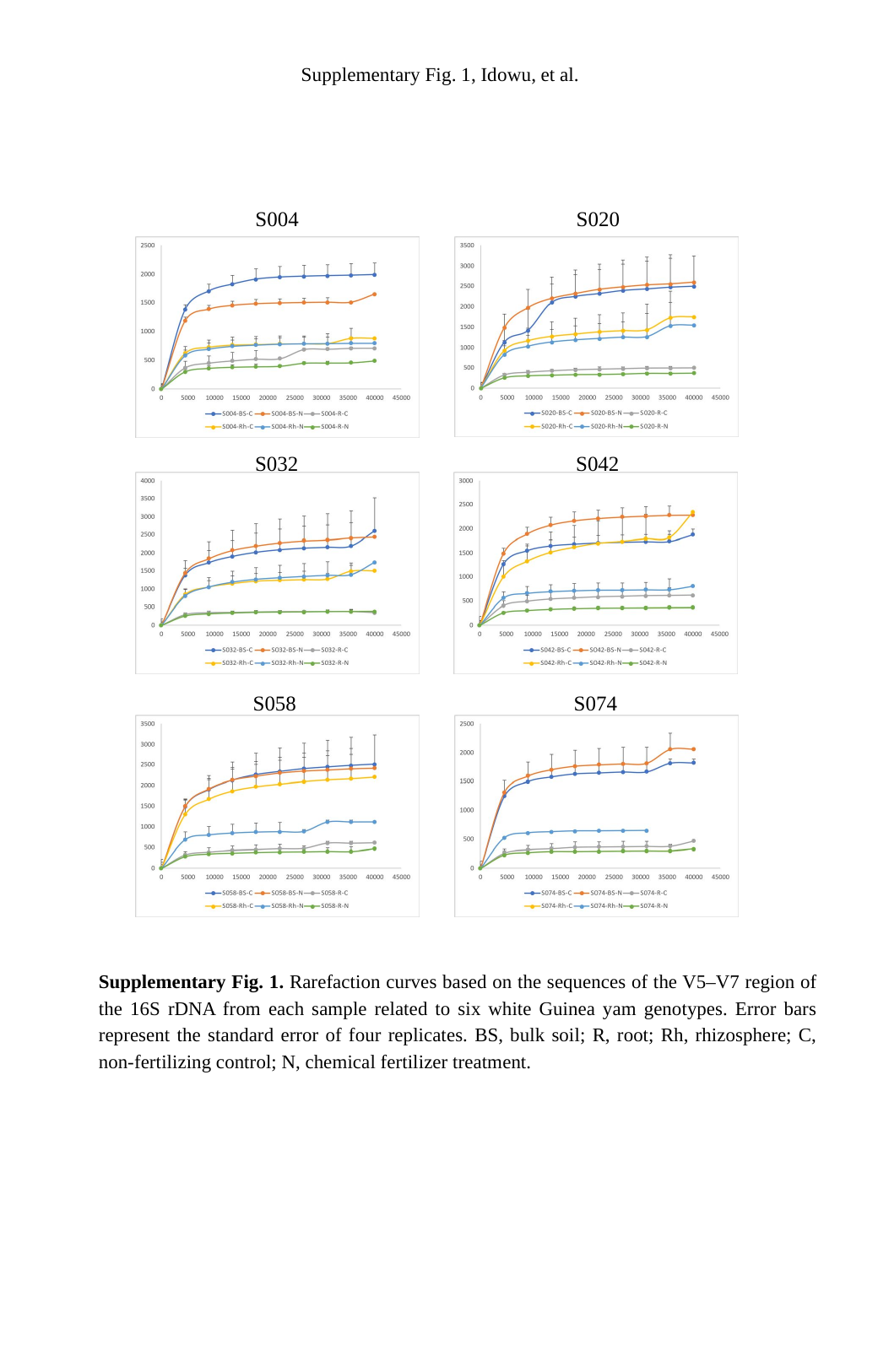

Supplementary Fig. 1, Idowu, et al.
S004
S020
S032
S042
S058
S074
Supplementary Fig. 1. Rarefaction curves based on the sequences of the V5–V7 region of the 16S rDNA from each sample related to six white Guinea yam genotypes. Error bars represent the standard error of four replicates. BS, bulk soil; R, root; Rh, rhizosphere; C, non-fertilizing control; N, chemical fertilizer treatment.
